# Effects of Cholecystokinin (CCK) on Gut Motility in the Stomachless Fish Ballan Wrasse (*Labrus bergylta*)

**DOI:** 10.3389/fnins.2019.00553

**Published:** 2019-06-07

**Authors:** Hoang T. M. D. Le, Kai K. Lie, Justine Giroud-Argoud, Ivar Rønnestad, Øystein Sæle

**Affiliations:** ^1^Feed and Nutrition, Institute of Marine Research, Bergen, Norway; ^2^Department of Biological Sciences (BIO), University of Bergen, Bergen, Norway

**Keywords:** Cholecystokinin, gut motility, wrasse (*Labridae*), contraction, stomachless, spatio temporal map

## Abstract

Cholecystokinin (CCK) is well-known as a key hormone that inhibits stomach emptying and stimulates midgut motility in gastric species. However, the function of CCK related to gut motility in agastric fish, especially in fish with a short digestive tract such as ballan wrasse, remains unknown. Here we present a detailed description of the spatio-temporal quantification of intestinal motility activity *in vitro* comprising the complete intestinal tract in ballan wrasse. We show that CCK modulates intestinal motility, having multiple effects on motility patterns depending on location in the gut and types of contractions. CCK reduced propagating contractions in the foregut, but it increased both non-propagating and propagating contractions in the hindgut. CCK also altered the direction of propagating contractions, as it reduced anterograde ripples and slow propagating contractions. The velocity of propagating contractions was slowed down by CCK. CCK also reduced the amplitude of standing contractions and ripples, but it did not alter the amplitude of slow propagating contractions. The presence of CCKA receptor antagonist modulated the motility responses of ballan wrasse intestines when exposed to CCK. We also showed that CCK reduced the intestinal length and stimulated motility to empty the gallbladder. Based on our findings we hypothesize that CCK, mainly through the CCKA receptor, modulates non-propagating and propagating contractions to optimize digestion and absorption and regulate the intestinal evacuation in ballan wrasse. We also found evidence that the modulation of intestinal motility by CCK is different in agastric fish from that in gastric vertebrates. We suggest that this is an evolutionary adaptation to optimize digestion without a stomach.

## Introduction

Gastrointestinal motility is an essential function of digestive and absorptive processes of the gut, as it is required for breakdown of food particles by mixing them with digestive enzymes and propelling intestinal contents along the gut for absorption of nutrient and elimination of unabsorbed particles (Chang and Leung, 2014). Gut motility is brought about by the contraction and relaxation of smooth muscles in the gastrointestinal tract and is classified into two main categories: (i) non-propagating contractions such as segmentation (mixing) and (ii) propagating contractions such as peristalsis and migrating motor complexes (MMCs) (Holmgren and Olsson, 2009). Gut motility in vertebrates is modulated by the autonomic nervous system (mainly by the enteric nervous system), gastrointestinal hormones, and by the appearance of food (Olsson and Holmgren, 2001; Campbell, 2009).

Cholecystokinin (CCK) is a key hormone in the control of digestion and serves as an anorexigenic factor assisting to terminate a meal in vertebrates. Levels of CCK mRNA increased after feeding while fasting reduced CCK expression in intestine and brain in platy *Xiphophorus maculatus* (Pitts and Volkoff, 2017) and in winter skate *Raja ocellata* (MacDonald and Volkoff, 2009). The roles of CCK in digestion include inhibition of gastric emptying rate and gastric secretion, and stimulation of gallbladder and intestine contractions and pancreatic lipase secretion (Aldman and Holmgren, 1987; Andrews and Young, 1993; Schjoldager, 1994; Olsson et al., 1999; Guilloteau et al., 2006; Volkoff, 2016).

CCK exerts it biological functions through its receptors, named Cholecystokinin A Receptor (CCKAR) and Cholecystokinin B Receptor (CCKBR) in mammals (Noble et al., 1999). In mammalian species, differences in the tissue-dependent distribution support different functional involvement of the two receptors. Based on the ubiquitously expression of CCKAR in the gastrointestinal tract, pancreas, gallbladder, and pyloric sphincter, it has been proposed that this CCK receptor is involved in gastrointestinal functions. In contrast, the CCKBR is mainly found in the mammal brain and is part of the regulation of anxiety, analgesia, learning, memory, and dopamine-related behaviors (Staljanssens et al., 2011). CCKBR is also found in a few types of cells in gastric mucosa, pancreas, and gastrointestinal tract and its functions in mammals are involved in gut development and enzyme secretion rather than gut motility (Guilloteau et al., 2006). In fish, the tissue distribution of the CCK receptors have been investigated in a few species such as Atlantic salmon (*Salmon salar*) (Rathore et al., 2013), yellowtail *Seriola quinqueradiata* (Furutani et al., 2013), and goldfish (Tinoco et al., 2015). However, a full understanding of the CCK receptors role in gastrointestinal functions is still lacking. Only two reports describe the function of CCK receptors, one in goldfish (Tinoco et al., 2015) and one for Siberian sturgeon (Zhang et al., 2017). These studies showed a contribution of CCKAR in the intestinal contractile response, while the CCKBR showed less involvement in digestive function. However, these studies only investigated the involvement of CCKAR in contraction force and the applied methods did not allow for detailed description and analysis of motility patterns.

The ballan wrasse is an agastric fish species of which the stomach has been lost during evolution both anatomically and functionally. This species has thus lost typical gastric genes like pepsin and the proton pumps, but also ghrelin, the only known orexigenic hormone produced in the digestive-tract (Lie et al., 2018). CCK, which is a hormone that in gastric species inhibits gastric contractions in order to regulate the passage of ingesta along the digestive tract at a proper rate, is also identified in ballan wrasse (Lie et al., 2018). However, the function of CCK and its receptors on intestinal motility in agastric fish remain largely unknown.

Gastrointestinal motility has been quantified using a range of analytical approaches including: changes in muscle tension, intraluminal pressure, membrane potential, and force of contractile response from smooth muscle (Lee, 1960; Costa and Furness, 1976; Kocylowski et al., 1979; Fioramonti et al., 1980; Sarna, 1986). Previous studies have presented movement activity at single or multiple positions along the gut and only showed the overall sense of motility patterns, for instance the force of contractions (Kiliaan et al., 1989; Clements and Rees, 1998; Velarde et al., 2009, 2010; Gräns et al., 2013; Tinoco et al., 2015). However, examination of gut motility also requires an understanding of the behavior of the whole intact intestine with spatial and temporal distributions of motility patterns along the different intestinal regions/segments. The motility patterns and contraction types in the fish intestine have been described for short intestinal segments in shorthorn sculpin (*Myoxocephalus scorpius*) (Brijs et al., 2014, 2016), rainbow trout (*Oncorhynchus mykiss*) (Brijs et al., 2017), halibut (Rønnestad et al., 2000), dogfish (*Scyliorhinus canicula)* (Andrews and Young, 1993), and zebrafish (*Danio reio*) (Holmberg et al., 2003, 2004, 2006, 2007). In fishes, there is a knowledge gap in how gut motility is involved in the regulation of gut evacuation in the different anatomical parts of the digestive system. For instance, the pyloric sphincter serves a role to regulate chyme flow propelling from the stomach to the intestine at a proper rate for optimal digestion in gastric fishes (Olsson, 2011; Olsson and Holmgren, 2011); however, what is responsible for regulating evacuation rate between the anterior intestine (which receives intact food from esophagus) and posterior intestine (which receives the semi-digested contents from the anterior part and continues the digestion) in agastric fish which lacks the stomach and thus, pyloric sphincter? The aim of this study was to describe the physiological impact of CCK on gut motility with the presence/absence of CCK-receptor antagonists in the complete intestinal tract. The secondary aim was to describe the general motility patterns in ballan wrasse intestine. To achieve this the spatio-temporal mapping method was adjusted so it could be done in R. The changes in motility patterns for the intestinal tract in ballan wrasse juveniles were examined using a mathematical model that quantified the correlation between spatial (location on the intestine) and temporal (time and velocity) distribution of contractions along the whole intestine based on data acquisition of video recording.

## Materials and Methods

### Animal and Tissue Preparation

Ballan wrasse juveniles were supplied by a commercial fish farm (Marine Harvest Labrus, Øygarden, outside Bergen, Norway). The fish were reared in accordance with the Norwegian Animal Welfare Act of 12 December 1974, no. 73, §§22 and 30, amended 19 June 2009. The facility has a general permission to rear all developmental stages of *Labrus berggylta*, license number H ØN0038 provided by the Norwegian Directorate of fisheries (https://www.fiskeridir.no/English). Fish were nursed in 3 m^3^–tanks in a temperature-controlled room (around 14°C) under a 24:0 h light:dark photoperiod and fed every 15 min. with a commercial pellet diet. Fish weighing 15–20 g were transferred from the Marine Harvest farm to the Institute of Marine Research (Bergen, Norway) laboratory and were kept at conditions identical to the nursing station for 1 day prior to running the experiments.

**Figure 1:**
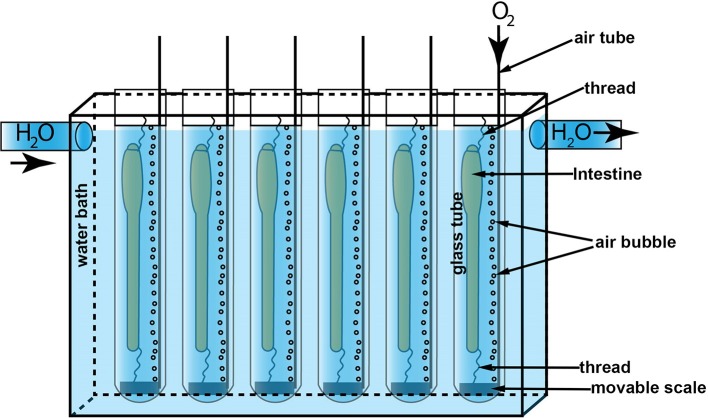
The *in vitro* experiment setup. The water bath (201 × 124 × 50 mm) was circulated with 14°C water pumped from a thermal chamber. Six glass tubes (*Φ* 20 mm) containing 25 mL Ringer's solution were immersed in the water bath. An air tube was mounted to each glass tube to supply oxygen to the solution. A scale was attached to the anal end of the intestines with surgical thread and the oral end of intestine was closed and attached to a hanger. This kept the intestine straight for measuring intestinal diameter but also allowed for longitudinal contractions.

The fish were anesthetized in 0.05 mg/mL tricaine methanesulfonate (MS222) dissolved in sea water prior to euthanasia and removal of the intestine. The eviscerated intestine included esophagus and anus with the surrounding skin, leaving the whole intestine intact. Eviscerated intestines (5–8 cm length) were immediately immersed in Ringer's solution according to Rønnestad et al. (2000) with some modifications (in mM: NaCl, 129; KCl, 2.5; MgCl_2_, 0.47; CaCl_2_, 1.5; NaHCO_3_, 20.2; and NaH_2_PO_4_, 0.42). The surrounding tissues like mesenteries and fat deposits were carefully removed, and the luminal content was gently flushed out. The prepared intestines were rapidly mounted in individual glass tubes containing 25 mL of Ringer's solution at 14°C, aerated with 95% O_2_ and 5% CO_2_. The intestines were carefully stretched out longitudinally inside the tube with the oral opening closed and the anus open. However, the mounted intestines were given enough slack to allow changes in length but also allowing for automated measurements of intestinal diameter. The anterior part of the intestine was attached with a thread tied around the esophagus and connected to a steel wire at the top of the incubation tube. The posterior part of the intestine was attached with surgical thread to flap of skin next to the anus and a small scale of 0.6–0.8 g depending on the size fish (see Figure 1 and Supplementary Table 1). The mounted intestines in the glass tube had an acclimatization period (15 min.) before the main treatments started, which is described in detail for each of the two experiments below.

### Drugs and Experimental Protocol

#### Experiment 1—Qualitative Test for Effects of Cholecystokinin (CCK) on Gut Motility

**Figure 2:**
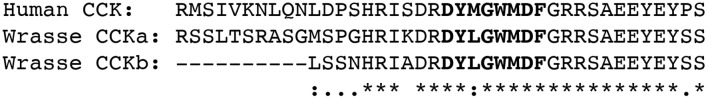
Alignment of the human CCK preproprotein (NP 000720.1) with the two CCK preproprotein isoforms from ballan wrasse CCKa (ENSLBEG00000007237) and CCKb (ENSLBEG00000024541). The CCK-8 fragment is highlighted in bold. Asterisk (^*^) indicates positions which have a single, fully conserved residue, colon (:) indicates conservation between groups of strongly similar properties—scoring >0.5 in the Gonnet PAM 250 matrix and period (.) indicates conservation between groups of weakly similar properties—scoring = <0.5 in the Gonnet PAM 250 matrix.

To investigate the effect of CCK on gut motility and identify the optimal concentration of CCK for Experiment 2, we qualitatively examined changes in motility patterns in intestines exposed to CCK at different concentrations. Based on the study in the agastric species, goldfish (Tinoco et al., 2015) and the similarity in the amino acid sequences between human and ballan wrasse (Figure 2), CCK peptide fragment 26–33 (CCK-8S, C2175, Sigma Aldrich) was used in Experiments 1 and 2. A theoretical physiological concentration of 20 pM was defined based on known CCK plasma levels found in rainbow trout (Jönsson et al., 2006) and a previous study in goldfish (Tinoco et al., 2015). Effect of five CCK-8S concentrations (0.2, 2, 20, 200 pM, and 2 nM) on motility was conducted in ballan wrasse intestines. The two lowest levels induced no change in motility patterns, thus we chose 20 pM as the minimum level and repeated the experiment with a five-level gradient. Each isolated intestine was exposed to freshly prepared CCK-8S, at one of five levels (20, 200 pM, 2, 20, and 200 nM) 30 min. (15 min. for acclimatization and 15 min. for control) after they were mounted into the glass tubes filled with aerated Ringer's solution. Instead of CCK-8S, distilled water was added to the control replicates. The experiment was registered with time lapse videos (see section Image Acquisition) immediately after the intestines were mounted in glass tubes for 50 min. (15 min. for acclimatization, 15 min. for control period, and 20 min. for CCK treatment). Spatio-temporal (ST) maps were constructed for 35 min. (15 min. for control period + 20 min. for CCK treatment period). The experiment was run in triplicate.

#### Experiment 2—Effects of CCK on Gut Motility in the Presence of CCK-Receptor Antagonists

To understand how the presence of CCK-receptor antagonists influenced the effect of CCK on gut motility, intestines were incubated with blockers prior to exposure to CCK-8S. Based on the study in the agastric species, goldfish (Tinoco et al., 2015), devazepide (D3821, Sigma Aldrich), and L-365260 (L4795, Sigma Aldrich) were used as antagonists to block the CCKA or CCKB receptors, respectively. After a 15-min. acclimatization period, the intestines were incubated with an antagonist, which was freshly dissolved in DMSO (DMSO - D8418, Sigma Aldrich), for 10 min. Isolated intestines were incubated with devazepide (CCKA receptor antagonist) or L-365260 (CCKB receptor antagonist); the final concentration in the glass tubes of the antagonists were 1 μM and 0.3 % for DMSO. Intestines in the control group were incubated with 0.3 % DMSO (final concentration) in Ringer's solution. After a 10-min. incubation period, CCK-8S was added to the incubation medium to obtain the final concentration of 2 nM. The final levels of antagonist and incubation time were designed based on the study by Tinoco et al. (2015) and our preliminary trials where 5, 10, 20, 30, and 60 min. of incubation of ballan wrasse intestines with the antagonists). The 2-nM concentration was chosen based on the results in Experiment 1 where the intestines induced a clear response to this level of CKK-8S in Ringer's solution. Experiment 2 was conducted on 4 fish for CCKAR-antagonist, and 5 fish for each CCKBR-antagonist/None-antagonist). The experiments were registered with time lapse videos (see section Image Acquisition) immediately after the intestines were mounted in glass tubes for 35 min. (15 min. for acclimatization, 10 min. for antagonist and/or DMSO incubation, and 10 min. for CCK treatment period). Motility patterns were analyzed for 20 min. (10 min. for antagonist and/ or DMSO incubation + 10 min. for CCK treatment period).

In order to examine effect of CCK on motility of gallbladder, the gallbladders containing bile were carefully removed from anesthesized ballan wrasse and immediately immersed in Ringer's solution. After a 20-min. acclimatization, gallbladders were exposed to 2 nM CCK-8S. The experiment was conducted with four gallbladders in 1 h for filming time lapse video.

### Image Acquisition

The incubator (201 × 124 × 50 mm) was fitted with six glass tubes. To ensure equal image quality and background a LED flood light aperture (15,000 lux) (Aputure Amaran AL-528W) covering the whole field of view (238 × 190 mm) was installed behind the incubator. A time lapse image series of intestines were captured during the experiment using a camera (Nikon DS-Fi3) with a macro lens (Nikon, AF Micro-Nikkor 60 mm f/2.8D), at a resolution of 1,024 × 768 pixels. The capture of the time lapse series was controlled with the NIS-Elements Confocal 4.51.01 software and captured 3.5 frames s^−1^ for the duration of 50 min. for Experiment 1 and 35 min. for Experiment 2. Six intestines were processed in parallel in each video (Figure 1).

### Gray-Scale Spatio-Temporal (ST) Map Construction

A test analysis was carried out on 20-min. videos of three fish to define the optimal interval of time lapse images for constructing ST maps and identifying contractions prior to running the main analysis. The ST maps and motility patterns were constructed and analyzed at 5 interval levels of 3.5, 1.2, 0.7, 0.35, and 0.02 frames s^−1^. This analysis showed that motility patterns on the ST maps and parameters of contractions evaluated in the videos at 3.5 and 1.2 frames s^−1^ were not different. However, the ST maps and contraction parameters were changed in the analysis in the videos with an interval ≤ 0.7 frames s^−1^. Hence, all analyses in the present study were examined in videos with an interval at 1.2 frame s^−1^.

#### Measuring Intestinal Diameter

**Figure 3:**
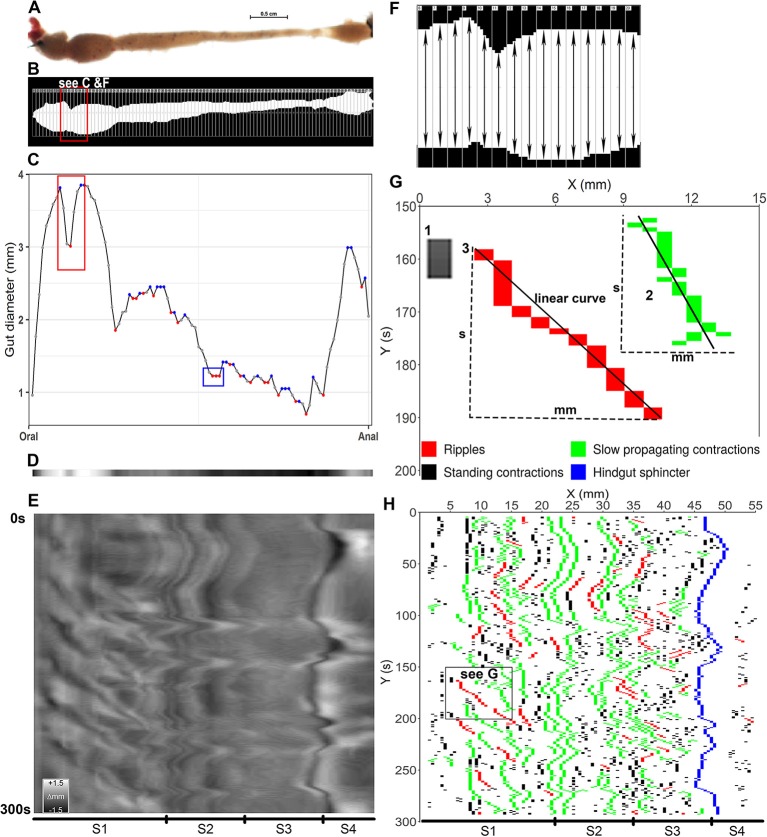
Converting video frames to ST maps and identifying contractions. **(A)** a video frame shows the ballan wrasse intestine which was transferred to binary color and drawn hundreds of regions of interest (ROI) in to measure intestinal diameter at each pixel **(B)**. **(C)** diameter values at each pixel along the intestine in **(B)** were displayed as dots in gray, blue and red colors; and the curve that connects the dots shows the dynamic change of the diameters from the anterior (oral) to posterior (anal) intestine. The blue dots show the local maximum values and the red dots for the local minima. The red rectangular shape in panel **(C)** shows the change of diameter values of the intestinal section in **(F)** where the red dot shows the smallest value of a ROI compared to those (gray dots) of the adjacent ROIs. In blue rectangular shape in panel **(C)** the first red dot was defined as a local minimum and the two next dots had a similar diameter to the first red dot, hence three of them were defined as contractions. **(D)** a gray band was constructed from the values of diameter in **(C)** as the lowest in black and the highest in white. **(E)** all frames in a trimmed 300 s video (Supplementary Video 2) were converted to gray bands to build up a gray-scale ST map on which the vertical direction showing the time and the horizontal direction showing the 4 segments (S1–S4) of intestine; the calibration bar shows changes in diameter as grayscale (Δmm). **(F)**, detail of an intestinal section showing a contraction; each rectangle with a number on the left corner was a ROI with its own ID (RoiID) to measure the distance between upper and lower edge of the white area inside (intestinal diameter). **(G)**, a successful contraction as an object on the binary map, was identified as either an isolated rectangle (1) or a cluster of rectangles connecting each other (2,3). Width (mm) represents for propagating distance, height (s) for duration, and slope and r-squared of the linear curves are used to identify contraction type, propagating direction and velocity. (1) A standing contraction; (2) A slow propagating contraction moves anterograde direction; 3, a ripple contraction propagates anterograde direction. **(H)**, a binary ST map was plotted from local minima selected on each frame (see **C**) shows tempo-spatial distribution of three contraction types along the four segments (S1–S4, Segments 1–4) of the ballan wrasse intestine.

Every third frame was subtracted from the original videos (3.5 frames s^−1^) to obtain 1.2 frame s^−1^ videos and calibrated for length and time before measuring intestinal diameter using Nis-Element software. A threshold for intensity was manually selected, which covered the whole intestinal area on each frame (Figures 3A,B). Background noise, generated by air bubbles and equipment accessories, were removed using the “restrictions” functions in Nis-Element software where the size of the intestine is defined based on pixel recognition and the program removes items in images appearing outside of the intestine. Rectangular regions of interest (ROI of 0.5 ^*^ 12 mm, width ^*^ height) were placed in stacks on each image series covering the whole length of the intestine (Figures 3B,F) using the function “draw ROI” in the Nis-Element software. The width of the ROIs was set to 0.5 mm to cover the intestinal diameter at each pixel according to D'Antona et al. (2001) and Brijs et al. (2014). The height (12 mm) was set according to the width of the video frame. The ROIs were applied automatically on the time lapse frames to generate a diameter-matrix which is a numeric matrix of intestinal diameter along intestine over time (see Supplementary Table 2 for an example of numeric matrix of intestinal diameter). Each ROI measured the diameter of the intestine based on pixel recognition on each of the frames analyzed, thus each matrix consists of intestinal diameters along the intestine in one direction and the diameters over time in the other. The diameter-matrix consists a number of sub-matrices, each of which represents the diameter along intestine on a video frame at a time point, namely frame-matrix (Supplementary Table 2).

#### Gray-Scale ST Map Plotting

A gray-scale spatio-temporal (ST) map (e.g., Figure 3E and Supplementary Figures 1A1–A3) represents the dynamic changes in values of diameter along the intestine over time as gray color. The gray-scale ST map consists of a number of single rows of pixels (Figure 3D) each of which is converted from the image on each video frame as the black color represented the minimum diameter and the white color for the maximum diameter. To generate the gray-scale ST map, average diameter during the recorded period was firstly calculated and subtracted from the diameter of each point on each frame to normalize the diameter. To smooth the ST maps a linear interpolation method was used according to Hazewinkel (1990). Nine values were calculated and inserted between each pair of diameters at two adjacent points along the intestine to interpolate the diameter-matrices. The normalized and interpolated numeric matrices were plotted to generate the gray-scale ST maps using the ggplot2 (geom_raster, scale_fill_gradient) package in R (version 3.4.2 released 2017-09-28) within R (studio) interphase (version 1.1.383) for Windows. The gray-scale ST maps were used to evaluate the changes in motility patterns in Experiments 1.

### Definition of Motility Patterns

#### Extraction of Contractions From Diameter-Matrices

Figure 3F shows the detail of an intestinal section with a distinct contraction. The intestinal position where a contraction is occurring is defined as the ROI that covers the smallest distance between the upper and lower edges of the white area compared to the adjacent ROIs (Figure 3F). A value that is less than other near values in a series of numbers is a local or relative minimum according to Garrett (2008) (red dots in Figure 3C). Based on this definition, the contractions were defined by extracting the local minima of a diameter at the threshold = 1 (red dots in Figure 3C) from every frame-matrix in the diameter-matrix. All ROIs that were near and have a similar diameter to the ROI covering the local maximum were also defined as a contraction. The amplitude of contractions was calculated as the delta of each pair of minimum and maximum. The frame-matrix then presented the defined contractions with their amplitude values, and where contractions were not registered NA (Not Available) were used (see Supplementary Table 3). A binary-matrix (Supplementary Table 3) consists of frame-matrices which present the amplitude of contractions and NA values was used to construct the binary ST maps (e.g., Figure 3H and Supplementary Figures 1B1–B3) and analyzed types and parameters of contractions.

#### Binary ST Map Plotting

To display the distribution of contractions along the intestine over time, excluding other information from the gray-scale ST map, a binary ST map (e.g., Figure 3H and Supplementary Figures 1B1–B3) was generated. The binary-matrices which present values of the defined contractions were used to construct the binary ST maps without the linear interpolation using the ggplot2 (geom_raster, scale_fill_gradient) package in R.

#### Classification of Motility Patterns

A successive contraction, as an object on the binary ST maps (Figure 3H), was identified as either an isolated rectangle (no. 1, Figure 3G) or a cluster of rectangles connecting with each other (no. 2–3, Figure 3G). In the binary-matrix, each contraction was defined as a sub-matrix which is made up of connected cells of numeric values, namely contraction-matrix (Supplementary Table 4). The contraction-matrix consists of the intestinal position (where on the intestine) the contraction occurs in mm measured from the top, time (when the contraction occurs - s), and values for the amplitude. The initiation site was defined as the value of the intestinal location in the numeric value cell which had the smallest value of time in the contraction-matrix (see “initiation site” in Supplementary Table 4). Propagating distance (in mm) is defined as the delta between maximum and minimum values of the intestinal position in the contraction-matrix. The intestinal position value of the initiation site and propagating distance (in mm) were expressed as the % of the entire intestine length. Duration of contraction was the difference (delta) between the maximum and minimum values of time in the contraction-matrix.

Contractions were classified into three types (motility patterns): standing contractions, ripples, and slow propagating contractions. Contractions which propagate a distance equal to or <1.0 mm were determined as standing contractions (no. 1 in Figure 3G & black pixels in Figure 3H). Contraction-matrices for contractions which have a propagating distance longer than 1.0 mm were analyzed for ripples (no.3 in Figure 3G and red pixels in Figure 3H) and slow propagating contractions (no.2 in Figure 3G and green pixels in Figure 3H). A linear correlation model was applied to find the regression between intestinal position (response vector) and time (a series of terms which specifies a linear predictor for intestinal position). Ripples were defined as contractions which have a coefficient of determination (*r*^2^) of the linear curve ≥0.8, based on the description of D'Antona et al. (2001) and Brijs et al. (2014). Slow propagating contractions, which propagate at a slow velocity, were defined with *r*^2^ < 0.8. Propagating velocity (mm s^−1^) was the absolute value of the slope of the linear curve. Propagating direction for ripples and slow propagating contractions were also identified from the slope, as slope > 0 for anterograde (oral toward anal) direction and slope < 0 for retrograde (anal toward oral) direction.

### Gene Expression and Sequence of CCK Receptors

In order to confirm the presence or absence of the CCK receptors along the different parts of the ballan wrasse intestine, we extracted normalized read counts (GSE93191, https://www.ncbi.nlm.nih.gov/geo/query/acc.cgi?acc=GSE93191) from a recently published study on ballan wrasse intestine (Lie et al., 2018). The previous mentioned report investigated the expression of genes related to nutrient absorption and metabolism in four segments along the ballan wrasse intestine. The normalized read counts were re-analyzed in Qlucore Oimcs Explorer 3.2 (Qlucore AB, Lund, Sweden).

### Data Analysis

The effects of CCK on motility patterns were qualitatively analyzed based on the changes on the gray_scale ST maps for 15 min. before and 20 min. after exposing the intestines to CCK in Experiment 1—effects of CCK on gut motility. For the general description of motility patterns (contraction types), we used the data collected from 21 fish: 6 fish from Experiment 1 (2 random fish for each of the two lowest CCK concentrations and the control); 9 fish from Experiment 2 (3 random fish from each treatment group), and 6 fish from the preliminary trials on empty intestines.

For Experiment 2, the effects of CCK with the presence of its receptor antagonists on motility patterns were defined based on the changes in contraction parameters compared with those prior to CCK administration. In this study, we used the definition of four intestinal segments in ballan wrasse according to the morphological description in Le et al. (2019, Figure 1). The morphologically defined intestinal segments were characterized in ten intestines, and the ratio of each segment of the total intestinal length were set based on the average of the ten characterized intestines. Average ratios of segment length/total intestine length for Segment 1, Segment 2, Segment 3, and Segment 4 are 0.39, 0.23, 0.23, and 0.15, respectively. Frequencies of contractions (contractions per min. on every mm length of intestine—cpm) in intestinal segments were calculated based on the site of initiation of contractions (Supplementary Table 5). The propagation orientation was presented as the proportion of contractions, propagating in either anterograde or retrograde direction, to the total sum of contractions.

We hypothesized that although contractions of the same type have the same physiological functions, their impact on the gut function may vary depending on their properties (e.g., amplitude and velocity). However, the parameters (amplitude, distance, duration, and velocity) were continuous values with an exponential distribution to which the comparison of average/median between treatments will be dominated by the large number of observations of small values. Thus, we used a mathematical method to classify the values of these parameters into three categories of low/short (value ≤ first quartile), medium (first quartile < value < third quartile), and high/(value ≥ third quartile) (Supplementary Table 6). Proportions of contractions for each category were then calculated. Based on results in Experiment 1, as the intestines responded more or less immediately after administration of CCK and the effect lasted <20 min., we examined effects of CCK on motility patterns within 10 min. before and 10 min. after the intestines were exposed to CCK. The frequencies and the proportions of contractions in each category for parameters were examined within the 10 min. before and 10 min. after CCK administration. Ratios of contraction parameter values measured after and prior to exposure to CCK were calculated and transformed to log_2_ to minimize the wide ranges of ratios (the ranges of ratios were wide due to the great variation between individual intestines). The log_2_(ratio)s were compared to 0 using one-sample *t*-test. The log_2_(ratio)s that were equal to 0, lower than 0, and >0 were considered as no change, a decrease, or an increase, respectively, in contraction parameters post-exposure to CCK. One-way ANOVA test followed by Tukey *post-hoc* test was used for analysis on data of length reduction of ballan wrasse intestine and frequency of three contraction types. Because of the large variation between individual intestines, 90% confidence interval was used instead of the more conservative 95%.

For the description for parameters of the three contraction types, a Kruskal–Wallis test followed by dunnTest (FSA package) was used to compare the median for amplitude, duration and velocity values between the three types of contractions, with *p* < 0.05 as statistical threshold. Changes in diameter is expressed as percent of average diameter and changes in intestinal length is expressed in relation to total intestinal length to be able to compare these parameters between individuals within treatments.

## Results

### CCK Receptors in Wrasse Intestine

**Figure 4:**
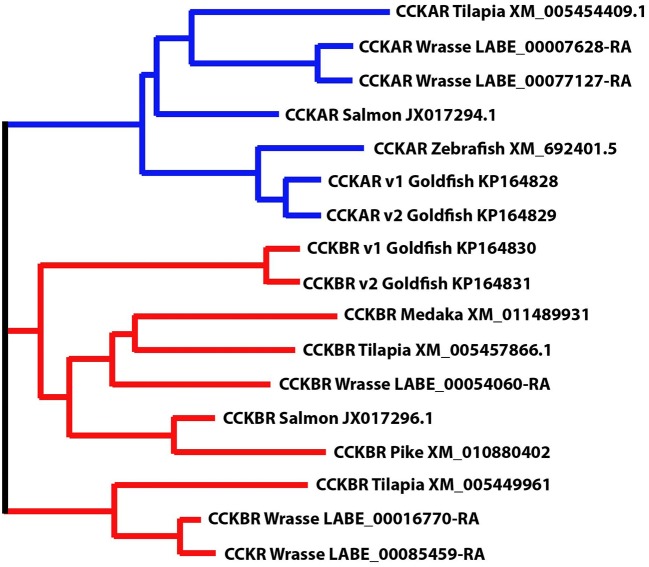
Phylogenetic position of ballan wrasse CCK receptors (CCKAR and CCKBR). The figure shows the phylogenetic relationship between the annotated CCK-receptor nucleotide sequences in tilapia, salmon, zebrafish, goldfish, medaka and pike to wrasse, generated using the Multiple Sequence Comparison by Log- Expectation (MUSCLE) tool.

Five CCK-receptor like genes were identified in the wrasse genome (European Nucleotide Archive accession number: PRJEB13687, http://www.ebi.ac.uk/ena/data/view/PRJEB13687). Using phylogenetic clustering, two and three genes resembling CCKAR and CCKBR in fish was identified, respectively (Figure 4).

There was no significant difference in expression of the CCK receptors between the different segments (*p* > 0.05). Two of the transcripts, one for CCKAR (LABE_00007628) and one for CCKBR (LABE_00054060) were higher expressed compared to the others (Table 1).

**Table 1:** Expression of CCKA receptor (*cckar*) and CCKB receptor (*cckbr*) in the four intestinal segments of ballan wrasse.

**Gene ID**	**Gene name**	**Segment 1 (fpkm)**	**Segment 2 (fpkm)**	**Segment 3 (fpkm)**	**Segment 4 (fpkm)**
LABE_00007628	cckar	4.7 ± 3.1	12.4 ± 7.7	16.2 ± 5.9	19 ± 21.5
LABE_00077127	cckar	0.8 ± 1	2.9 ± 2.9	2.1 ± 2.4	3.1 ± 3.8
LABE_00054060	cckbr	21.7 ± 18.8	7.5 ± 6.3	5.6 ± 3.5	18.3 ± 8.2
LABE_00016770	cckbr	1 ± 1.4	0.3 ± 0.7	1.7 ± 1.6	4.1 ± 6.1
LABE_00085459	cckbr	0 ± 0	0.3 ± 0.5	0.2 ± 0.4	1.7 ± 1.8

*The expression values are normalized RNAseq read counts expressed as fragments per kilobase of transcript per million (fpkm). Segment 1 is the anterior bulbous which is wider than the rest. Segment 2 and 3 are the first and second half of the intestine between the bulbous and the hindgut. Segment 4 is the hindgut. Descriptive figure of the ballan wrasse digestive tract can be found on Figure 1 in Le et al. (2019)*.

### Properties of Motility Patterns in Intestine Ballan Wrasse Juvenile

Measurement of width along the intestines of ballan wrasse juveniles showed dynamic changes in the intestinal diameter which reflected smooth muscular driven contractions and relaxations of the gut (Supplementary Videos 2–5). Depending on propagating distance and velocity, the contractile activity was classified as standing contractions, ripples, or slow propagating contractions.

#### Standing Contractions

**Figure 5:**
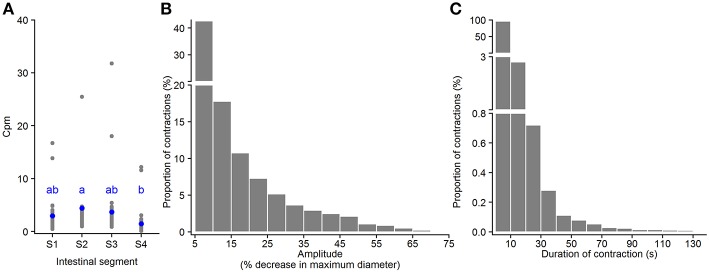
Motility parameters for standing contractions in intestine of ballan wrasse juveniles. **(A)** Frequency of standing contractions (contractions per min. per mm gut length, cpm) in the four intestinal segments (gray dots for individual values and blue dots for mean value calculated from 21 fish); letters denote difference between segments (ANOVA, Tukey *post-hoc* test). **(B)** Amplitude of standing contractions was converted from mm to a relative value (%) in relation to the maximum diameter of the intestinal position where the contraction occurred. **(C)** Duration of standing contractions. **(B,C)** present histogram of data that was pooled from 21 fish. S1–S4, Segments 1–4 of the ballan wrasse intestine.

Standing contractions, a form of non-propagating contractions, were identified as contractions with a very short propagating distance (≤ 1mm) (black pixels in Figures 3G,H and in Supplementary Figures 1B1–B3). The standing contractions were observed in all four segments, with 91.5% in the three first segments (S1–S3) and 8.5% in the hindgut (based on all standing contractions from 21 fish). This contraction type occurred at 0.4 – 16.7 (2.3) [min – max (median)] contractions per min. per mm length of intestine (cpm) in Segment 1 (anterior bulbous); 1.0 – 41.1 (3.2) cpm in Segment 2; 0.9 – 31.8 (2.6) cpm in Segment 3; and 0.1 – 12.2 (0.9) cpm in Segment 4 (hindgut). The frequency of standing contractions was not different between the three first segments (S1–S3), but this contraction type occurred at a higher rate in Segment 2 than in Segment 4 (*p* = 0.02, ANOVA, Tukey *post-hoc* test) (Figure 5A). The amplitude of standing contractions varied from 5.0 to 74.5%, with a median of 11.1% decrease in maximum diameter of the point where the contraction occurred. The standing contractions with an amplitude ≤ 10% decrease in maximum diameter was the dominant contraction type (45.1% of all the contractions, Figure 5B). The standing contractions prolonged from 0.3 to 127.0 s with most of these contractions (95.9%) were of short duration (<10 s). Standing contractions with a duration of 10–20 s accounted for 2.7%, and very few (1.4%) contractions lasted longer than 20 s (Figure 5C).

#### Ripples

**Figure 6:**
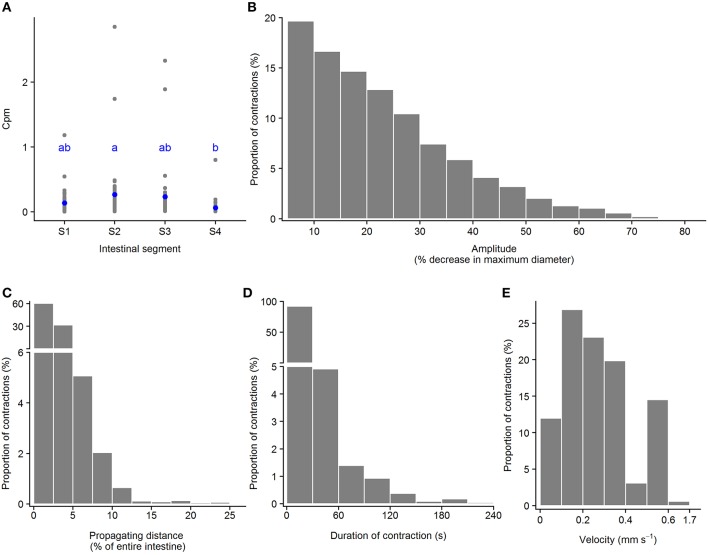
Motility parameters for ripples in intestine of ballan wrasse juveniles. **(A)** Frequency of ripples (contractions per min. per mm gut length, cpm) in the four intestinal segments (gray dots for individual values and blue dots for mean value calculated from 21 fish); letters denote difference between segments (ANOVA, Tukey *post-hoc* test). **(B)** Amplitude was converted from mm to a relative value (%) in relation to the maximum diameter of the intestinal position where the ripple occurred; **(C)** Propagating distance; **(D)** Duration; and **(E)** Propagating velocity of ripples. **(B–E)** present histogram of data that was pooled from 21 fish. S1–S4, Segments 1–4 of the ballan wrasse intestine.

Ripples are a subtype of propagating contractions that is characterized by rhythmic contractions of the circular muscle (red pixels in Figures 3G,H and in Supplementary Figures 1B1–B3); according to (D'Antona et al., 2001). Based on the total number of the ripples in 21 intestines analyzed, 28.7, 34.8, 30.1, and 6.3% of the ripple contractions were initiated at the first (S1), second (S2), third (S3) segment, and the hindgut (S4), respectively. The frequency of ripples was from [min – max (median)] 0.004 – 1.183 (0.075) cpm in Segment 1; 0.007 – 2.852 (0.137) cpm in Segment 2; 0.01 – 2.33 (0.10) in Segment 3; and 0.01 – 0.80 (0.03) in Segment 4. The frequency of ripples had no difference between the first three segments (S1–S3), but ripples occurred in Segment 4 at lower rate than Segment 2 (*p* = 0.04, ANOVA, Tukey *post-hoc* test) (Figure 6A). The ripple contractions propagated in either the anterograde 12.5 – 82.4 (54.1) [min – max (median)] or the retrograde direction 17.6 – 87.5 (45.9) (% of the total number of ripples occurring in the individual intestine). The amplitude of ripples, decrease in the maximum diameter of the point where the contraction occurred, varied from 5.0 to 79.9%, with a median at 19.8% (Figure 6B). The ripples covered a wide range of distances, from 1.1 to 23.0%, with a median at 2.1% of the entire intestinal length (Figure 6C). The ripples which propagated a distance ≤ 2.5% of the entire intestine length dominated with 60.2% of the total number of ripples. The duration of ripples was between 0.6 and 238.0 s with a median at 6.3 s and 92% of the ripples lasted for 30 s or less (Figure 6D). The ripple contractions propagated at various velocities, from 0.002 to 1.694 mm s^−1^, with the median 0.239 mm s^−1^ (Figure 6E).

#### Slow Propagating Contractions

**Figure 7:**
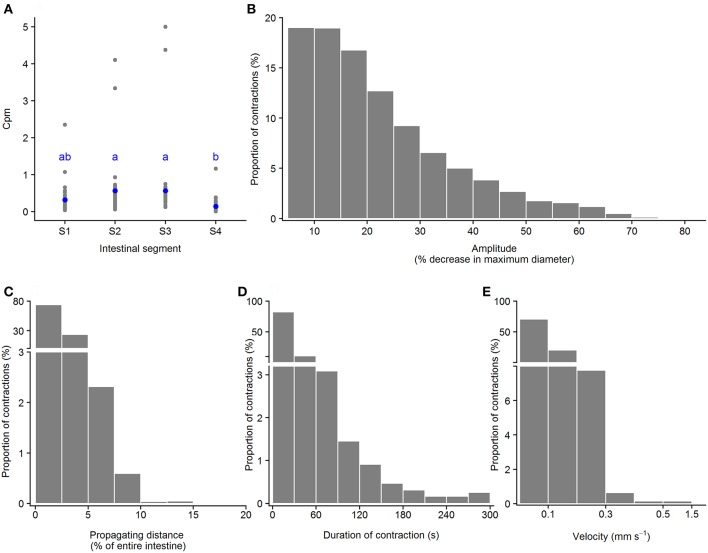
Motility parameters for slow propagating contractions in intestine of ballan wrasse juveniles. **(A)** Frequency of slow propagating contractions (contractions per min. per mm gut length, cpm) in the four intestinal segments (gray dots for individual values and blue dots for mean value calculated from 21 fish); letters denote difference between segments (ANOVA, Tukey *post-hoc* test). **(B)** Amplitude was converted from mm to a relative value (%) in relation to the maximum diameter of the intestinal position where the slow propagating contractions occurred; **(C)** Propagating direction; **(D)** Duration of contractions; **(E)** Propagating velocity of slow propagating contractions. **(B–E)** present histogram of data that was pooled from 21 fish. S1–S4, Segments 1–4 of the ballan wrasse intestine.

Another type of propagating contractions in ballan wrasse intestines was the slow propagating contractions (green pixels in Figures 3G,H and in Supplementary Figures 1B1–B3). Slow propagating contractions were recorded in all four segments. Of all analyzed contractions in 21 intestines in this category, 28.7% were initiated in Segment 1, 32.9% in Segment 2, 31.6% in Segment 3 and 6.8% in Segment 4. Slow propagating contractions occurred at a rate of [min – max (median)] 0.04 – 2.35 (0.23) cpm in Segment 1; 0.1 – 4.1 (0.4) cpm in Segment 2; 0.1 – 5.0 (0.3) in Segment 3; and 0.01 – 1.16 (0.09) in Segment 4. Segments 2 and 3 were found to present this contraction type at a higher frequency than Segment 4 (*p* = 0.01, ANOVA, Tukey *post-hoc* test) (Figure 7A). 29.9 – 67.4 (53.0) [min – max (median)] % of these contractions propagated in an anterograde direction and 32.6 – 70.1 (47.0) % in the retrograde propagation direction. The amplitude of the slow propagating contractions ranged from 5.0 to 85.4%, with a median of 18.6% decrease in maximum diameter of the point where the contraction occurred (Figure 7B). The propagating distance of the slow contractions ranged from 1.1 to 17.0%, with a median at 2.0% of the entire intestinal length (Figure 7C). Propagating duration also varied from 0.3 to 296.1 s with median at 11.7 s, of which durations equal to or <30 s were dominant (82.3% of the contractions) (Figure 7D). Velocity of slow propagating contractions ranged from 0.001 to 1.471 mm s^−1^ with a median at 0.057 mm s^−1^. 70.7% these contractions had a propagation speed equal to or <0.1 mm s^−1^ and the rest had a speed from 0.100 to 1.471 mm s^−1^ (Figure 7E).

The Kruskal-Wallis test showed that ripples had higher amplitude than other contraction categories (ripples > slow propagating contractions > standing contractions, Table 2). Slow propagating contractions lasted a longer time than the ripples and standing contractions (Table 2). The two types of propagating contractions had similar propagating distance (*p* = 0.19), but ripples propagated at a faster velocity than slow propagating contractions (*p* < 0.0001). Standing contractions had higher frequency than other contraction types (ANOVA, *p* < 0.0001).

**Table 2:** Median amplitude, duration, and velocity of the three contraction types.

	**Standing contractions**	**Ripples**	**Slow propagating contractions**	***p*1**	***p*2**	***p*3**
Amplitude (% decrease in maximum diameter)	11.1	19.8	18.6	<0.0001	< 0.0001	<0.0001
Distance (% of entire intestine length)	–	2.1	2.0	–	0.19	–
Duration (s)	0.9	6.3	11.7	<0.0001	< 0.0001	<0.0001
Velocity (mm s^−1^)	–	0.239	0.057	–	< 0.0001	–

*P-values for Kruskal-Wallis test followed by dunnTest are presented in column p1 for standing contractions vs. ripples, p2 for ripples vs. slow propagating contractions, p3 for slow propagating contractions vs. standing contractions. Data from three pools of three contraction type which were made up from 21 fish; with n = 82383 for the pool of standing contraction, 4522 for ripple, and 10251 for slow propagating contraction. The parameters were compared between the three pools of contraction types*.

### Experiment 1—Which Concentrations of CCK Induced Alteration in Gut Motility?

**Figure 8:**
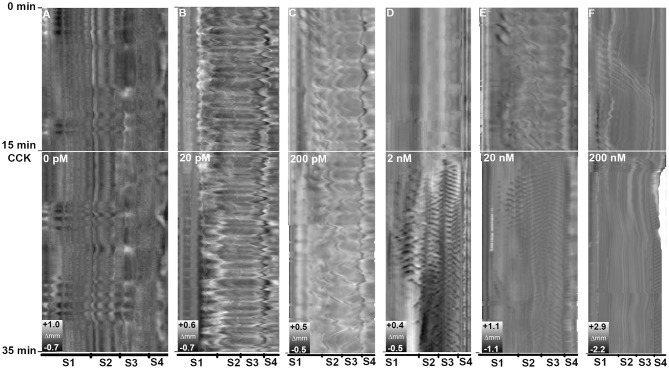
Effects of concentration CCK-8S on motility in ballan wrasse intestine. **(A)** control without adding CCK-8S to the medium; Intestine was exposed to CCK-8S in the medium at concentration at **(B)** 20 pM, **(C)** 200 pM, **(D)** 2 nM, **(E)** 20 nM, and **(F)** 200 nM. The white line marks when CCK was added to the medium. Each concentration has three replicates and the figure shows an example from the Experiment 1.

CCK-8S induced effects on the motility pattern *in vitro* at concentrations of 2, 20, and 200 nM. The intestines exposed to 20 and 200 pM CCK and the controls showed no change in motility pattern (Figures 8A–C). The motility pattern representing ubiquitous standing contractions obviously changed in the intestines exposed to 2 and 20 nM CCK medium, as more frequent ripples occurred from posterior part of the first segment to the third segment (Figures 8D,E). Beside the increase in ripples, a reduction in gut length occurred in intestines exposed to 20 nM CCK-8S. The 200 nM CCK-8S concentration induced frequent ripples in the hindgut and reduced the intestinal length, but abated the ripples and slow propagating contractions in the three first segments (Figure 8F).

### Experiment 2—Effects of CCK on Gut Motility With the Presence of CCK-Receptor Antagonists and DMSO

#### Standing Contractions

**Figure 9:**
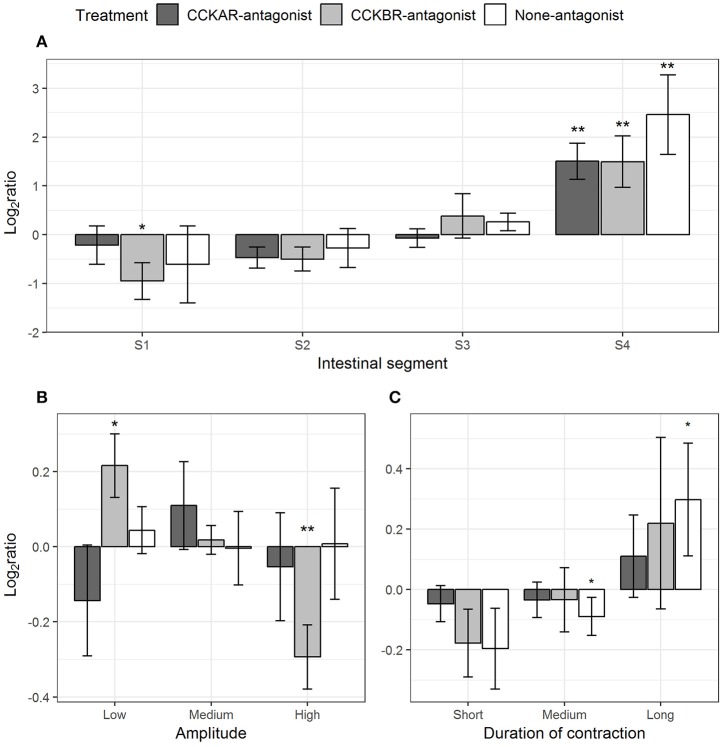
Effect of CCK-8S with presence of CCK-receptor antagonists on standing contractions. CCKAR-antagonist: intestines were pre-incubated with devazepide, CCKBR-antagonist: intestines were pre-incubated with L-365260, and None-antagonist: intestines were pre-incubated with DMSO as a control prior to exposing intestine to CCK-8S. The three plots show log_2_ of ratios of contraction parameters recorded after to before exposing intestine to CCK-8S: **(A)** for frequency of standing contractions measured in four intestinal segments (S1, S2, S3, and S4); **(B)** for proportion of standing contractions which had a low, medium or high amplitude; **(C)** for proportion of standing contractions which prolonged for a short, medium, or long duration. Bars are presented with mean ± SEM (*n* = 4 for CCKAR-antagonist, *n* = 5 for each CCKBR-antagonist/none-antagonist). Asterisk (^*^) denotes the statistically significant differences of ratio values compared with 0 (one sample *t*-test, ^*^for 0.05 ≤ *p* < 0.1 and ^**^p < 0.05).

The presence of CCK-receptor antagonists affected frequency and amplitude of standing contractions induced by CCK-8S. In intestines pre-incubated with CCKBR antagonist, frequency of standing contractions decreased in Segment 1 (one-sample *t*-test, *p* = 0.06) and increased in Segment 4 (one-sample *t*-test, *p* = 0.04) (CCKBR-antagonist, Figure 9A). In intestines pre-incubated with either CCKAR antagonist or DMSO (control), frequency of standing contraction in the three first segments had no significant change after adding CCK-8S but the frequency increased in the last segment (S4) (one-sample *t*-test, *p* < 0.005) (S4, CCKAR-antagonist and None-antagonist, Figure 9A).

The inhibition of CCKBR led to reduced amplitude of standing contractions, i.e., CCK-8S increased the proportion of low-amplitude standing contractions (one-sample *t*-test, *p* = 0.06) whereas it decreased the proportion of high-amplitude contractions (one-sample *t*-test, *p* = 0.02) (CCKBR-antagonist, Figure 9B). No change in amplitude of standing contractions was observed in the CCKAR-antagonist and control treatments. CCK-8S seemed to increase the duration of standing contractions; i.e., the proportion of medium-duration contractions decreased (one-sample *t*-test, *p* = 0.09) and the proportion of long-duration contractions increased (one-sample *t*-test, *p* = 0.08) (None-antagonist, Figure 9C). The effect of CCK-8S on duration of standing contractions was not clear in the intestines pre-incubated CCKR antagonists (CCKAR-antagonist and CCKBR-antagonist, Figure 9C).

#### Ripples

**Figure 10:**
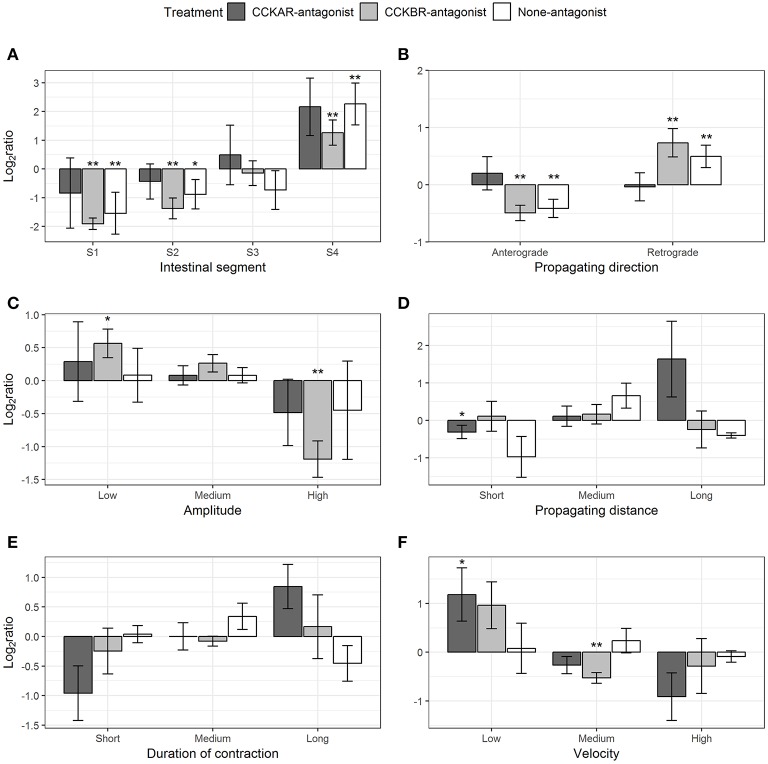
Effect of CCK-8S with presence of CCK-receptor antagonists on ripples. CCKAR-antagonist: intestines were pre-incubated with devazepide, CCKBR-antagonist: intestines were pre-incubated with L-365260, and None-antagonist: intestines were pre-incubated with DMSO as a control prior to exposing intestine to CCK-8S. The six plots show log_2_ of ratios of contraction parameters recorded after to before exposing intestine to CCK-8S: **(A)** for frequency of ripples measured in four intestinal segments (S1, S2, S3, and S4); **(B)** for proportion of ripples which propagated with an anterograde or retrograde direction; **(C)** for proportion of ripples which had a low, medium or high amplitude; **(D)** for proportion of ripples which propagated for a short, medium, or long distance; **(E)** for proportions of ripples which prolonged for a short, medium, or long duration; **(F)** for proportion of ripples which propagated at a low, medium or high velocity. Bars are presented with mean ± SEM (*n* = 4 for CCKAR-antagonist, *n* = 5 for each CCKBR-antagonist/none-antagonist). Asterisk (^*^) denotes the statistically significant differences of ratio values compared with 0 (one sample *t*-test, ^*^ for 0.05 ≤ *p* < 0.1 and ^**^p < 0.05).

Pre-incubation with CCKR antagonists had effects on a range of parameters of ripples evoked by CCK-8S. CCK-8S reduced the ripples in Segment 1 (one-sample *t*-test, *p* = 0.04) and Segment 2 (one-sample *t*-test, *p* = 0.02) and increased ripples in Segment 4 (one-sample *t*-test, *p* = 0.01) (None-antagonist, Figure 10A). The effects of CCK-8S on intestines pre-incubated with CCKBR antagonist had a similar pattern to that in the None-antagonist group. CCK-8S reduced the ripples in the two first segments (Segments 1 and 2) (one-sample *t*-test, *p* < 0.05) but it increased the frequency of these contractions in the hindgut (Segment 4) (CCKBR-antagonist, Figure 10A). CCK-8S did not affect this contraction type in the four segments in intestines pre-incubated with CCKAR antagonist (CCKAR-antagonist, Figure 10A).

CCK-8S regulated the propagating direction of ripples; i.e., the proportion of retrograde ripples increased after adding CCK-8S (one-sample *t*-test, *p* = 0.03) (None-antagonist, Figure 10B). This effect of CCK-8S on propagating direction of ripples was also seen in CCKBR-antagonist treatment (one-sample *t*-test, *p* = 0.04) (CCKBR-antagonist, Figure 10B). The propagating direction of ripples was not altered by CCK-8S in the CCKAR-antagonist treatment.

CCK-8S reduced the amplitude of ripples in the intestines pre-incubated with CCKBR antagonist, i.e., it increased the proportion of low-amplitude ripples (*p* = 0.06) and decreased the proportion of high-amplitude contractions (*p* = 0.01) (CCKBR-antagonist, Figure 10C). The amplitude of ripples in CCKAR-antagonist and None-antagonist treatments were not affected by CCK-8S.

The effect of CCK-8S with the presence of its receptor antagonists on propagating distance was not clear. CCK-8S reduced the proportion of short-distance ripples (*p* = 0.01) but it did not alter the medium- and long-distance ripples in intestines in the CCKAR-antagonist treatment. The propagating distance in intestines in the CCKBR-antagonist and None-antagonist treatments were not influenced by CCK-8S (Figure 10D). Exposing intestines to CCK-8S after incubating them with CCK-receptor antagonists or DMSO did not induce alterations in the propagating duration of ripples (Figure 10E).

There was no change in velocity of ripples after adding CCK-8S in the control group (None-antagonist, Figure 10F). However, pre-incubation of intestine with CCKBR antagonist before adding CCK-8S seemed to reduce the velocity of ripples. The proportion of low-velocity ripples seemed to increase and that of medium-velocity decreased (*p* = 0.004) after adding CCK-8S to the organ bath (CCKBR-antagonist, Figure 10F). Presence of CCK-8S also reduced the velocity of ripples, as the proportion of low-velocity ripples increased in intestines pre-incubated with CCKAR antagonist after exposing intestines to CCK-8S (*p* = 0.08) (CCKAR-antagonist, Figure 10F).

#### Slow Propagating Contractions

**Figure 11:**
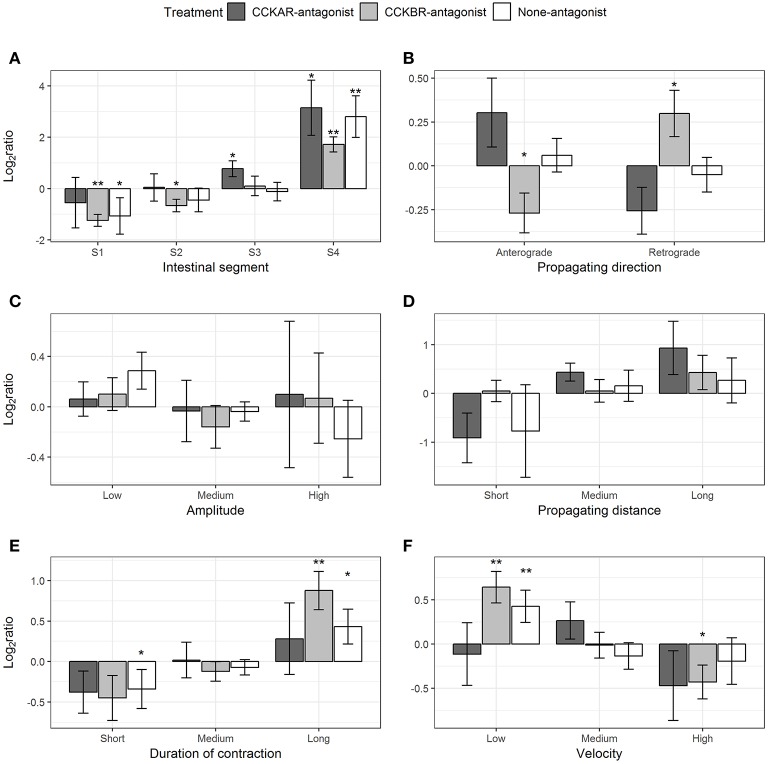
Effect of CCK-8S with presence of CCK-receptor antagonists on slow propagating contractions. CCKAR-antagonist: intestines were pre-incubated with devazepide, CCKBR-antagonist: intestines were pre-incubated with L-365260, and none-antagonist: intestines were pre-incubated with DMSO as a control prior to exposing intestine to CCK-8S. The six plots show log_2_ of ratios of contraction parameters recorded after to before exposing intestine to CCK-8S: **(A)** for frequency of slow propagating contractions measured in four intestinal segments (S1, S2, S3, and S4); **(B)** for proportion of slow propagating contractions which propagated with an anterograde or retrograde direction; **(C)** for proportion of slow propagating contractions which had a low, medium or high amplitude; **(D)** for proportion of slow propagating contractions which propagated for a short, medium, or long distance; **(E)** for proportions of ripples which prolonged for a short, medium, or long duration; **(F)** for proportion of slow propagating contractions which propagated at a low, medium or high velocity. Bars are presented with mean ± SEM (*n* = 4 for CCKAR-antagonist, *n* = 5 for each CCKBR-antagonist/none-antagonist). Asterisk (^*^) denotes the statistically significant differences of ratio values compared with 0 (one sample *t*-test, ^*^for 0.05 ≤ *p* < 0.1 and ^**^ for *p* < 0.05).

Most of the parameters of slow propagating contractions induced by CCK-8S were affected by the presence of CCKR antagonists, except for amplitude and distance. CCK-8S inhibited slow propagating contractions in Segment 1 (one-sample *t*-test, *p* = 0.09), but it induced more in the Segment 4 (*p* = 0.02) (None-antagonist, Figure 11A). The effects of CCK-8S on this contraction type in the CCKBR-antagonist treatment had the same pattern as the None-antagonist treatment, where it inhibited these contractions in Segments 1 (*p* = 0.005) and 2 (*p* = 0.05) and stimulated more contractions in Segment 4 (*p* = 0.004) (CCKBR-antagonist, Figure 11A). In intestines pre-incubated with CCKAR antagonist, CCK-8S increased slow propagating contractions in the Segments 3 (*p* = 0.08) and 4 (*p* = 0.06) but it did not alter motility in the two first segments (S1–S2) (CCKAR-antagonist, Figure 11A).

CCK-8S regulated the propagating direction of slow propagating contractions in the intestine pre-incubated with CCKBR antagonist. Exposing intestines to CCK-8S resulted in a decrease in the proportion of anterograde contractions (*p* = 0.07) and an increase in the proportion of retrograde contractions (*p* = 0.08) (CCKB-antagonist, Figure 10B). However, CCK-8S did not have an effect on propagating contractions when CCKAR antagonist was presented and in the control treatments (CCKAR-antagonist and None-antagonist, Figure 11B).

CCK and its receptors were not involved in regulation of amplitude and propagating distance of slow propagating contraction (Figures 11C,D). The duration of slow propagating contractions was prolonged by CCK-8S. The proportion of short-duration contractions was reduced in the None-antagonist treatment (*p* = 0.07) and that of long-duration contractions was increased in the CCKBR-antagonist (*p* = 0.01) and None-antagonist treatments (*p* = 0.07) (Figure 11E).

CCK-8S reduced the velocity of slow propagating contractions. In the None-antagonist treatment, the proportion of low-velocity contractions increased after adding CCK-8S to organ bath (*p* = 0.04). Also, in the CCKBR-antagonist treatment, CCK-8S increased the proportion of low-velocity contractions (*p* = 0.01) and reduced that of high-velocity contractions (*p* = 0.09) (CCKBR-antagonist, Figure 11F). The velocity of slow propagating contraction in the CCKAR-antagonist treatment were not altered by CCK-8S (Figure 11F).

#### Reduction in Gut Length

**Figure 12:**
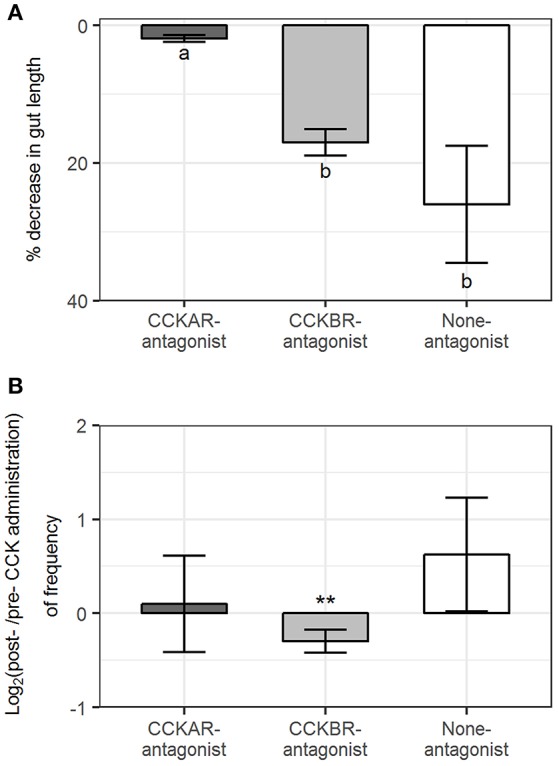
Effect of CCK-8S with presence of CCK-receptor antagonists on longitudinal contractions. CCKAR-antagonist: intestines were pre-incubated with devazepide, CCKBR-antagonist: intestines were pre-incubated with L-365260, and none-antagonist: intestines were pre-incubated with DMSO as a control prior to exposing intestine to CCK-8S. **(A)** decrease in length of intestine after being exposed to CCK-8S and **(B)** log_2_(ratio) of frequency of events for intestinal length reduction on intestine after to before being exposed to CCK-8S. Bars are presented with mean ± SEM (*n* = 4 for CCKAR-antagonist, *n* = 5 for each CCKBR-antagonist/none-antagonist). Different letters indicate statistically significant differences (*p* < 0.05; ANOVA, Tukey *post-hoc* test) among three treatments (CCKAR-antagonist, CCKBR-antagonist and none-antagonist) in **(A)**. ^*^Statistically significant differences of log_2_(ratio) values compared with 0 (one sample *t*-test, ^*^for 0.05 ≤ *p* < 0.1 and ^**^for *p* < 0.05) in **(B)**.

Intestines were mounted with flexibility to move in the organ baths, which enabled us to register the changes in intestinal length. CCKAR antagonist had an effect on reduction of the intestinal length induced by CCK-8S. Length reduction induced by CCK-8S in intestines in CCKAR-antagonist group (1.9 ± 0.5%) was smaller than those in CCKBR-antagonist (16.9 ± 1.9%) and None-antagonist groups (25.9 ± 8.5%) (ANOVA, *p* < 0.001) (Figure 12A). CCK-8S decreased the frequency of events for intestinal length reduction in the CCKBR-antagonist treatment (CCKB-antagonist, Figure 12B). However, the frequency of these events did not change after exposing intestines to CCK-8S in the rest treatments (CCKAR-antagonist and None-antagonist, Figure 11B).

### Effect of CCK on Gallbladder Motility

The gallbladders behaved differently between pre- and post-administration of CCK-8S. Waves of light contractions occurred in the bladder before the bladders were exposed to CCK-8S. Administration of CCK-8S into a bath with Ringer's solution induced a strong tonic contraction and consequently emptying of the bladder *in vitro* (Supplementary Video 1).

## Discussion

### An Alternative Way for Detailed Analysis of Motility Patterns

Here we present a refined method based on time lapse image analysis and spatio-temporal mapping that significantly improve the ability to quantify gastrointestinal motility. The principle of the spatio-temporal mapping method is based on changes in luminal diameter of the digestive tract over time visualized as heat maps. Types of motility, and the parameters of contractions such as propagating direction, distance, duration, velocity and frequency are identified and calculated directly relying on the spatio-temporal (ST) maps by using a series of different filters, thresholds and criteria to detect contractions (e.g., Brijs et al., 2014, 2017). Image analysis has previously been visualized in ST maps by different proprietary software packages/programs depending on research groups (D'Antona et al., 2001; Hennig et al., 2010; Brijs et al., 2014; Kendig et al., 2016). Our study presents an alternative method using R and NIS-Elements software to convert the changes on intestinal diameter into the ST maps and analyze the gut motility pattern. The gray-scale ST maps from Experiment 1 showed changes in motility patterns in isolated intestines incubated with different concentrations of CCK-8S. This suggests that the ST maps constructed by our method represent adequate information for changes in diameter along the intestine which allow to analyze movement patterns of whole intestine segments as the previous approaches have provided.

Gastrointestinal motility patterns have been extensively described in fishes and mammals using different classification systems (Holmgren and Olsson, 2011; Chang and Leung, 2014), the most common definitions of motility are the two main categories namely non-propagating and propagating contractions. Standing contractions, a non-propagating contraction type, were defined as contractions which propagates in a short distance <1mm in this study, based on the description by Cannon (1912), Chang and Leung (2014), and Huizinga et al. (2014). Ripples, one of the propagating contraction types, which have been described by D'Antona et al. (2001), were defined using linear correlation coefficient according to Brijs et al. (2014). In the recent study, the linear correlation coefficient method was also used to extract slow propagating contractions which propagate at a slow velocity and with longer duration compared to ripples (Brijs et al., 2014). As mentioned in the previous paragraph, all parameters of contractions/ motility patterns have been defined on ST maps based on gray color representing diameter values, using a ranges of software tools and plugins in previous studies (D'Antona et al., 2001; Hennig et al., 2010; Brijs et al., 2014). In the present study, we located the contractions using extraction of local minima from the diameter-matrix—the numeric matrices of intestinal diameter. All the parameters of contractions were directly extracted and analyzed from the diameter matrices using mathematical models, which prevent the objective limitation of visual evaluations of ST maps.

### Presence of CCKAR Antagonist Modulates the Response of Ballan Wrasse Intestine to CCK

In this study, we used CCK-8S to assess the role of CCK in ballan wrasse. CCK peptides are derived from proCCK in endocrine cells and neurons to produce varied-sized CCK molecules. The C-terminal octapeptide of CCK (CCK-8), a small CCK peptide, is mainly released by both the endocrine and neuronal cells in the gastrointestinal tract in tetrapods (Beinfeld, 2003; Rehfeld, 2017) and fish (Jensen et al., 2001; MacDonald and Volkoff, 2009; Pereira et al., 2015). The octapeptide CCK-8 has the highest activity in humans (Escrieut et al., 2002). Thus, CCK-8 has been commonly used in studies for effects of CCK on feed intake and digestion in mammals (e.g., Yamagishi and Debas, 1978; Raybould and Tache, 1988; Konturek et al., 1990; Schwizer et al., 1997). The CCKA receptor (CCKAR) has a greater affinity for sulfated than non-sulfated CCK whereas the binding ability of the B receptor (CCKBR) is not affected by sulfation of the tyrosine residue in CCK-8 (Dufresne et al., 2006; Staljanssens et al., 2011). Since the structure of CCK-8 is highly conserved through phylogeny, including fish (Johnsen, 1998; Murashita et al., 2006; Zhang et al., 2017), the commercial sulfated CCK has been used to study the function CCK peptides in appetite regulation and gastrointestinal motility (Olsson et al., 1999; Forgan and Forster, 2007; Tinoco et al., 2015; Zhang et al., 2017). Also, the wrasse CCK-8 has seven out of eight amino acids that are similar to those in human CCK-8 (Figure 2). For these reasons, CCK-8S was chosen to evaluate the effects of CCK and its receptors on intestinal motility in ballan wrasse.

In ballan wrasse, we identified five CCK-receptor genes corresponding to CCKAR and CCKBR in other teleosts. CCKAR has been reported to be involved in contractile response of fish intestine (Tinoco et al., 2015; Zhang et al., 2017). The CCKAR has been known to be more involved in digestion than the CCKBR in mammals (Thomas et al., 1979; Dufresne et al., 2006; Staljanssens et al., 2011; Rehfeld, 2017). The present study revealed that CCK induced a broad range of contraction parameters in intestines in CCKBR-antagonist and/or None-antagonist treatments, but these effects were absent in the CCKAR-antagonist treatment. This indicates that the presence of CCKAR antagonist reduced the effects of CCK on intestinal motility. However, the effects of CCK on a few contraction parameters (e.g., amplitude of ripples, propagating direction of slow propagating contractions, and frequency of events for intestinal length reduction) were recorded in the CCKBR-antagonist intestines but they were absent in the None-antagonist intestines. It may be secondary effects of the presence of CCKBR antagonist on the intestinal motility which should be tested in the future.

The present study shows the effect CCK with the presence of 0.3% DMSO (final concentration in medium) on the intestinal motility of ballan wrasse. The concentration of DMSO used in this study was in the middle of the accepted doses for cell culture [0.1–0.5% (Chen and Thibeault, 2013)]. Also, it was under the dose of DMSO which induces relaxation rabbit detrusor muscle must higher than 1% (Shiga et al., 2007). However, the potential effect of the low concentrations of DMSO on gut smooth muscle should be tested.

### How CCK Is Involved in Digestion in the Agastric Fish

CCK seems to modulate the intestinal evacuation to optimize digestion and absorption by regulating non-propagating and propagating contractions in ballan wrasse. CCK is involved in inhibiting foregut evacuation by reducing frequency and velocity of propagating contractions, which are involved in transferring gut contents distally along the digestive tract (Campbell, 2009; Chang and Leung, 2014). Also, CCK “modifies” standing contractions (i.e., CCK reduces the amplitude but prolongs the duration of standing contractions to act as a “physiological sphincter” (Chang and Leung, 2014) to allow gut contents to pass through the foregut at appropriate times. Moreover, CCK evoked more retrograde ripples and slow propagating contractions that may propel gut contents backward to foregut to complete the digestion. Thus, a slow evacuation rate of the foregut, together with the propulsion of gut contents backward to the anterior bulbous, may prolong the residence of digesta in Segment 1, the main site for digestion and absorption of nutrients in ballan wrasse, where up to 70% of macronutrients are absorbed (Le et al., 2019). As in other fish species, ballan wrasse has two CCK coding genes (A and B) of which *cckb* was mainly expressed in the most anterior part of the intestine (Segment 1) while the *ccka* was constituently expressed along the intestine (Lie et al., 2018), similar to its receptors as shown in the present study.

We hypothesize that the functions of CCK in modulation for gut motility and digestion in the agastric ballan wrasse may partly correspond to that in gastric species. In mammals, CCK has been demonstrated to inhibit the gastric contractions, and stimulate motility of small intestine and gallbladder to proceed the digestion process (Thomas et al., 1979; Christoph, 1994; Grider, 1994; Schwizer et al., 1997; Chang and Wong, 2009; Rehfeld, 2017). In gastric fish, CCK had effects on modulation of gastric emptying, stomach motility (Olsson et al., 1999) and gallbladder motility (Aldman and Holmgren, 1987). Intact food enters stomach and is broken down to chyme (partly digested food mixed with GI-tract secreta) prior to portions of chyme is passed on to the small intestine for further digestion by pancreatic enzymes and consequent absorption of nutrients. Thus, CCK reduces gastric motility to prevent too much chyme to enter the intestine at once and increases motility of the intestine to move chyme along the intestine. However, in the agastric wrasse, intact food and not chyme that enters the intestine (i.e., anterior bulbous). Consequently, the pancreatic enzymes must break down intact food items, which requires more time than digestive processing of chyme. Thus, the motility (both non-propagating and propagating contractions) were inhibited in the anterior bulbous (Segment 1) by the presence of CCK. In contrast to the stimulation of midgut motility in gastric species, the motility of midgut (standing and slow propagating contractions in Segments 2 and/or 3) was either not affected or reduced (ripples in Segment 2), by CCK in the agastric ballan wrasse. Since the anterior bulbous in agastric fish is the analog to the small intestine in gastric fish (and other vertebrates), CCK serves different functions in this section in different species. We hypotheses that this is an adaptation to agastric digestion.

Ripples and slow propagating contractions are both propulsive patterns and they have similar propagating distances in ballan wrasse. However, ripples had the more or less similar amplitude compared to slow propagating contractions, whereas they are shallow contractions in shorthorn sculpin intestine (Brijs et al., 2014). This suggest that in ballan wrasse ripples may play a role in not only mixing/circulating thanks to the fast propagating velocity (Ehrlein et al., 1983; D'Antona et al., 2001; Hennig et al., 2010; Chen et al., 2013), but also propelling digesta, which is assisted by the high amplitude (Husebye, 1999; Kunze and Furness, 1999; Bharucha, 2012).

The presence of CCK peptide in the hindgut of ballan wrasse has been confirmed by Lie et al. (2018). We have also shown that CCK participates in regulating hindgut motility, as it induced more contractions of the three types in Segment 4. Increase in frequency of ripples in the guinea-pig colon correlated with the raise in volume of intraluminal fluid (Hennig et al., 2010). Moreover, Brijs and coworkers found that ripples were involved in stimulating water absorption, which might be a response to the increased drinking rate of saltwater fish in a hyper osmotic environment (Brijs et al., 2017). This suggests that the high level of fecal moisture in the hindgut (Le et al., 2019) may stimulate release of CCK; which increases ripples and standing contractions to mix and circulate the gut contents for water absorption. CCK exposure also increased slow propagating contractions in the hindgut (Segment 4) and prolonged the duration of this contraction type, which may stimulate defecation of waste products out of the intestine (Kellow, 1996) in ballan wrasse.

CCK also decreased the frequency of gut length reduction events in the ballan wrasse. A propagating contraction is a synchronization of circular muscle contraction and longitudinal muscle contraction in mammals (Burnstock, 1958; Yokoyama and North, 1983; Smith and Robertson, 1998) and fish (Brijs et al., 2014). The reduction of gut length may resemble the contractions of longitudinal muscle which have found to decrease in fed fish (Brijs et al., 2014). This suggests that presence of food in a digestive system stimulates releasing CCK (Smith and Gibbs, 1985; Moran, 2000) to suppress longitudinal contractions which contribute to prevent propulsion of intestinal contents to adjacent sections (Melville et al., 1975).

CCK induced strong tonic contractions in the gallbladder of ballan wrasse. Bile in the gallbladder is mixed by the waves of light contractions. Presence of lipid in the gastrointestinal tract stimulates enterocytes to secret CCK (McLaughlin et al., 1998; Beglinger and Degen, 2004); CCK then suppresses mixing movements and induces tonic contractions to pump bile (Spellman et al., 1979; Xu et al., 2008) from the bladder to intestinal lumen to form micelles and solubilize lipids.

## Conclusion

We have developed a new method using mathematical models to define motility patterns registered with time lapse images *in vitro*. Our method allows both qualitative visualization (ST maps) and quantitative description of the three types of contractions. The quantitative analysis in the agastric ballan wrasse show that CCK modulates intestinal motility through mainly CCKAR. CCK inhibits the propagating contractions (ripples and slow propagating contraction) in the foregut (Segment 1) and reduces the velocity and amplitude of these contractions most likely to prevent propelling gut contents to adjacent sections; but rather keeps the standing contractions mixing the gut contents to optimize the digestion and absorption of nutrients. Our findings suggest that CCK has a different strategy in the modulation of intestinal motility compared to that known in gastric vertebrates. We hypothesize that this is an evolutionary adaptation in ballan wrasse to optimize digestion without a stomach. Frequency of retrograde ripples and slow propagating contractions were increased by CCK to propel gut contents backwards to the foregut to complete digestion. CCK stimulates both non-propagating and propagating contractions in the hindgut, which might be involved in water absorption and removing waste products out of the intestine in ballan wrasse. CCK is involved in digestion *via* altering intestinal motility to optimize evacuation rate for optimal digestion and absorption and stimulating contractions in gallbladder to fill bile salt to the intestine in ballan wrasse.

## Ethics Statement

Ballan wrasse juveniles were supplied by a commercial fish farm (Marine Harvest Labrus, Øygarden, outside Bergen, Norway). The fish was reared in accordance with the Norwegian Animal Welfare Act of 12 December 1974, no. 73, §§22 and 30, amended 19 June 2009. The facility has a general permission to rear all developmental stages of Labrus berggylta, license number H ØN0038 provided by the Norwegian Directorate of fisheries (https://www.fiskeridir.no/English).

## Author Contributions

HL, IR, KL, and ØS: planning and preparing experiment. HL, KL, JG-A, and ØS: participated in carrying out experiment. HL, KL, JG-A, ØS, and IR: analysis. HL, KL, and ØS: writing draft version. HL, KL, IR, and ØS: editing.

### Conflict of Interest Statement

The authors declare that the research was conducted in the absence of any commercial or financial relationships that could be construed as a potential conflict of interest.
