# Supplementary material for: Effects of Cholecystokinin (CCK) on Gut Motility in the Stomachless Fish Ballan Wrasse (Labrus bergylta)
Source: Front Neurosci. 2019 Jun 7;13:553. doi: 10.3389/fnins.2019.00553 (PMC6568239; doi:10.3389/fnins.2019.00553)
Supplement: Supplementary file 9 [file Date_Sheet_1.docx]

Supplementary Material

Effects of cholecystokinin (CCK) on gut motility in the stomachless fish, ballan wrasse (*Labrus bergylta*)

Hoang T. M. D. Le^1,2*^, Kai K. Lie^1^, Justine Giroud-Argoud^1^, Ivar Rønnestad^2^, Øystein Sæle^1^

*** Correspondence:** Hoang T. M. D Le: Hoang.Le@hi.no

# Supplementary Tables

**Supplementary Table 1. Fish size and weight of scale mounted to intestine.**

| **Fish weight (g)** | **Scale (g)** |
| --- | --- |
| 15 – 16.9 | 0.6 |
| 17 – 18.9 | 0.7 |
| 19 – 20.9 | 0.8 |

**Supplementary Table 2.** **Diameter-matrix and frame-matrix**. A numeric matrix presents the intestinal diameter (in mm) along a ballan wrasse intestine in a 300 s video. The first row shows the position on the intestine where the intestinal is measured (Intestinal position). The first column shows the time points when the video frames are captured. From the second to the last rows, each row represents a series of diameter (in mm) along the intestine which are measured on intestine on frame at a time point of 0 s to 299.69 s. Each row of data is named a frame-matrix. *(Please see Table 1.XLSX file uploaded to Supplementary material)*

**Supplementary Table 3.** **Binary-matrix.** A matrix is constructed from the diameter matrix (S1 Table) by locating contractions using local maxima and minima extraction. The numeric cells in the matrix show the amplitude (% in change of relaxation diameter at the point where the contraction occurs). The coordinate values of the numeric cells (inherited the first row and first column) show the spatial (where on the intestine) and temporal (when) distribution of the contraction. The values of others cells, which show no contractions occur, are replaces by NA. *(Please see Table 2.XLSX file uploaded to Supplementary material)*

**Supplementary Table 4.** **Contraction-matrix**. The matrix on the left show the spatio-temporal distribution of a number of successful contractions. A successful contraction is defined as one or a number of numeric cells (blue-filled cells) which connect to each other (restricted in black-colored border). On the right table, examples of some contraction-matrices for a ripple, slow propagating contraction, and standing contraction which are extracted from the left table. Linear correlation coefficient (r-squared), slope of linear curve, distance, duration, type of contraction and propagating direction are calculated and defined for each contraction matrix. *(Please see Table 3.XLSX file uploaded to Supplementary material)*

**Supplementary Table 5. The intestinal segments for initiation site of contraction**

| Initiation site  (% of entire intestine length) - X | Intestinal segment |
| --- | --- |
| X ≤ 39 | Segment 1* (S1) |
| 39 < X ≤ 62 | Segment 2 (S2) |
| 62 < X ≤ 85 | Segment 3 (S3) |
| X ≥ 85 | Segment 4 (S4) |

* Segment 1 contains the anterior bulbous (Le et al 2019 <https://doi.org/10.3389/fmars.2019.00140>)

**Supplementary Table 6. Classification of contraction parameters**

| Parameter | Contraction type |  | Category |  |
| --- | --- | --- | --- | --- |
|  |  | Low/Short | Medium | High/Long |
| Amplitude (%) | Standing contraction | ≤ 7.2 | 7.2 – 21.0 | ≥ 21.0 |
|  | Ripples | ≤ 11.5 | 11.5 – 30.4 | ≥ 30.4 |
|  | Slow propagating contraction | ≤ 11.5 | 11.5 – 29.1 | ≥ 29.1 |
| Duration (s) | Standing contraction | ≤ 0.9 | 0.9 – 1.8 | ≥ 1.8 |
|  | Ripples | ≤ 2.7 | 2.7 – 13.5 | ≥ 13.5 |
|  | Slow propagating contraction | ≤ 7.1 | 7.1 – 23.4 | ≥ 23.4 |
| Distance (mm) | Ripples | ≤ 1.6 | 1.6 – 3.1 | ≥ 3.1 |
|  | Slow propagating contraction | ≤ 1.4 | 1.4 – 2.7 | ≥ 2.7 |
| Velocity  (mm s^-1^) | Ripples | ≤ 0.15 | 0.15 – 0.39 | ≥ 0.39 |
|  | Slow propagating contraction | ≤ 0.02 | 0.02 – 0.11 | ≥ 0.11 |

# Supplementary Videos

**Supplementary Video 1. Effect of CCK-8S on gallbladder motility.** The first part of video shows the activity of gallbladder before adding CCK-8S with the waves of contractions. The second part of video shows the response of gallbladder to CCK-8S (from addition of CCK onward), as the bladder wall contract to pump the bile liquid out the bladder lumen. The video is played at a speed of 30 times faster than the original speed (*Please see Video 1.MP4*)

**Supplementary Video 2 - 5. Examples of motility in empty intestines of ballan wrasse.** The video is played at a speed of 30 times faster than the original speed. The grey-scale spatio-temporal (ST) maps were constructed from the videos. *(Please see Video 2.MP4, Video 3.MP4, Video 4.MP4, and Video 5.MP4 files uploaded to Supplementary material)*

# Supplementary Figures


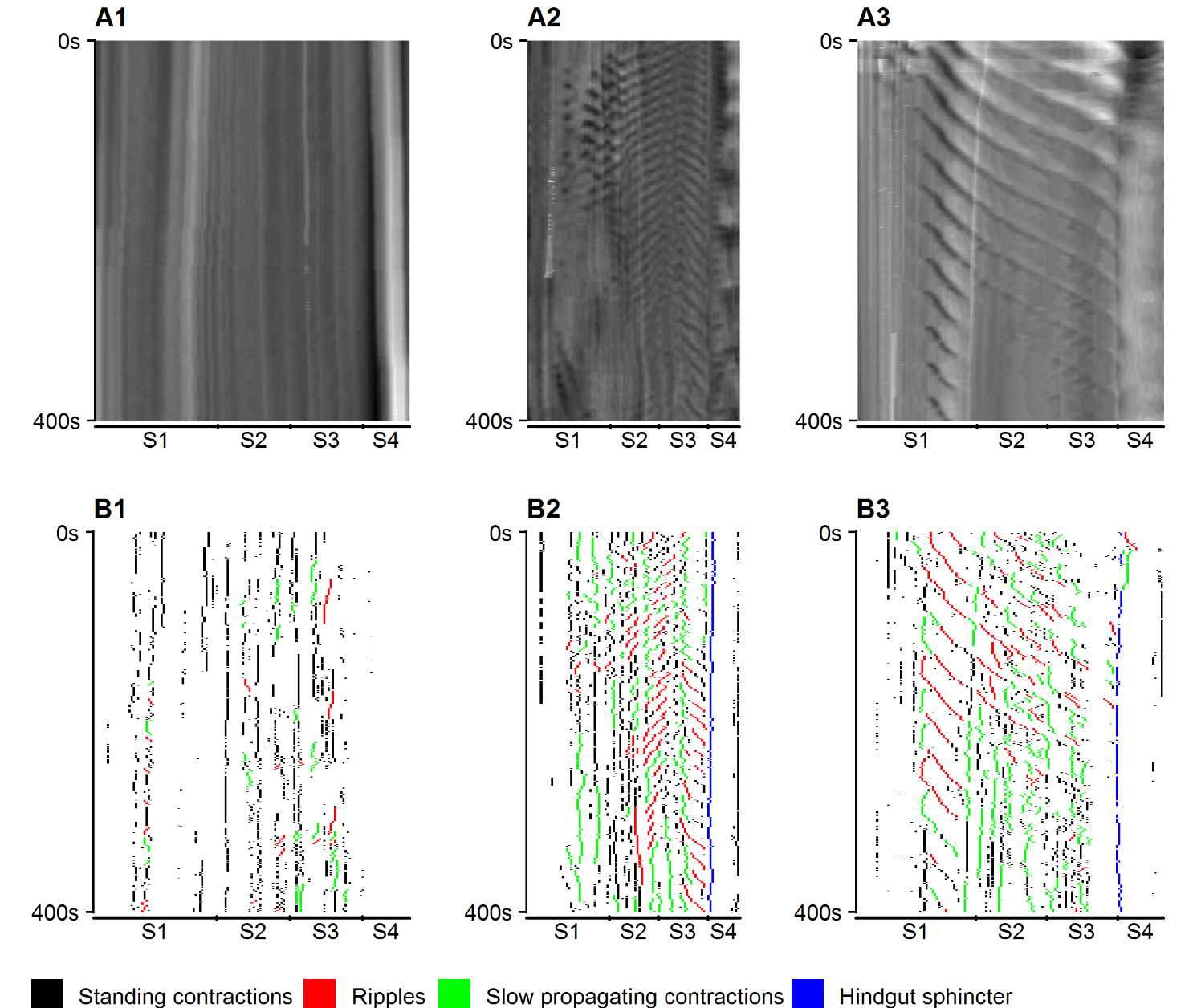


**Supplementary Figure 1. Grey-scale and binary spatio-temporal (ST) maps.** Three examples of motility patterns in empty intestines of ballan wrasse which have been showed in the **Supplementary Videos 2- 4**. A1 & B1 were constructed from **Supplementary Videos 3** (see ***Video 3.MP4*** *file*); A2 & B2 from **Supplementary Videos 4** (see ***Video 4.MP4*** *file*); and **Supplementary Videos 5** (see ***Video 5.MP4*** *file*). The motility of each intestines within 400 second was presented in the grey-scale maps (A1 – A3) on the first row and the three types of contractions exhibiting in each individual were showed in the binary maps in different colors (B1-B3, respectively). The hindgut sphincter was possible to be identified in intestines in A2 and A3, which was presented as blue color in the binary maps (B2 and B3)
